# Continuous veno-venous hemofiltration for treatment of enterovirus 71-induced fulminant cardiopulmonary failure: a case report

**DOI:** 10.1186/1752-1947-6-159

**Published:** 2012-06-26

**Authors:** Phuc Huu Phan, Hung Viet Dau, Son Thanh Chu, Thuy Bich Phung, Thang Van Pham, Tu Van Nguyen, Liem Thanh Nguyen

**Affiliations:** 1 The National Hospital of Pediatrics, 18/879 Lathanh Street, Hanoi, Dongda District, Vietnam

## Abstract

**Introduction:**

Fulminant cardiopulmonary failure is a severe complication of hand, foot and mouth diseases due to enterovirus 71 infection, with a high mortality rate. The treatment is mainly supportive with aggressive cardiopulmonary resuscitation. We report the use of continuous veno-venous hemofiltration in a patient with pulmonary edema and shock due to enterovirus 71 infection. To the best of our knowledge, this is the first report of the use of continuous veno-venous hemofiltration to successfully treat a patient with fulminant cardiopulmonary failure due to enterovirus 71 infection.

**Case presentation:**

A 36-month-old Asian girl presented to our hospital with pulmonary edema, refractory hypotension and severe cardiac dysfunction due to enterovirus 71 infection. In addition to the standard management and care, we performed continuous veno-venous hemofiltration to overcome refractory shock and our patient eventually made a full recovery. At a three-month follow-up, a full assessment revealed no neurological sequelae.

**Conclusions:**

In the management of patients with enterovirus 71-related fulminant cardiopulmonary failure, early continuous veno-venous hemofiltration may be considered as an alternative treatment to improve patient survival and to prevent severe neurological disabilities.

## Introduction

Outbreaks of hand, foot and mouth disease (HFMD) have occurred in Vietnam and other countries in the Western Pacific region with high morbidity and mortality over the last decades. In 2011, from the beginning of the year to December, 106,508 cases and 162 deaths were reported in Vietnam, with a high proportion associated with enterovirus (EV71) [1]. Fulminant cardiopulmonary failure (CPF), characterized by pulmonary edema, refractory shock and severe left ventricular dysfunction, is the most severe complication of EV71-associated HFMD. Most of the reported patients with EV71-induced pulmonary edema have deteriorated and died within 24 hours of hospitalization [2]. The pathogenesis of severe EV71 infection remains unclear. However, marked elevation of several cytokines and chemokines was noted in patients with brainstem encephalitis and pulmonary edema [3,4].

Current treatment for EV71-related CPF is mainly supportive with aggressive cardiopulmonary resuscitation. Several rescue therapeutic modalities, such as a left ventricular assist device and extracorporeal life support (ECLS), have been used for the treatment of EV71-related CPF with limited results [5]. Recently, Jan *et al*. reported a case series of 13 children with CPF due to EV71 who had undergone either extracorporeal left ventricular support or extracorporeal membrane oxygenation. Although 10 out of 13 patients were successfully weaned off ECLS, four out of five patients with hypotensive CPF died, and one survived with severe neurological sequelae. In addition, a number of complications occurred among patients on ECLS [5].

Over the last decades, with the application of continuous renal replacement therapy (CRRT), outcomes of seriously ill infants and children have improved. CRRT has been documented as effective treatment for patients with multiple organ failure due to severe sepsis and septic shock [6]. In addition, early and high-volume continuous veno-venous hemofiltration (CVVH) improved the hemodynamic status and cytokine removal in patients with systemic inflammatory response syndrome and septic shock [7,8]. However, the effectiveness and clinical experience in the use of this treatment modality in the management of children with EV71-related cardiopulmonary failure remains limited. Here, we report a child with pulmonary edema, refractory shock and left ventricular dysfunction due to EV71 infection who survived after emergency treatment with CVVH therapy.

## Case presentation

A previously healthy 36-month-old Asian girl was presented to us with two days of high fever and an erythematous rash and vesicles on her hand and mouth. The child did not have vomiting, diarrhea or cough. She had several myoclonic jerks per day. On admission to the department of infectious diseases at the National Hospital of Pediatrics in Hanoi, Vietnam, our patient had a temperature of 39°C per axilla. Her oxygen saturation was 99% in room air. Her noninvasive blood pressure (BP) was 116/77mmHg and her heart rate was 144 beats per minute. She appeared alert. An initial full blood count revealed a white blood cell (WBC) count of 10,900 cells/μL. A chest X-ray was normal. HFMD stage 2b was diagnosed and she received paracetamol and phenobarbitone for symptomatic treatment. Immunoglobulin (1g/kg) was given intravenously for 8 hours.

About 30 hours after admission, the child rapidly deteriorated with respiratory distress, tachycardia and reduced consciousness, and was admitted to the pediatric intensive care unit (PICU). She immediately required endotracheal intubation and positive pressure ventilation support. A pinkish frothy secretion was noted in the endotracheal tube. A physical examination at the time of PICU admission revealed a temperature of 40.0°C per rectum, a heart rate of 210 beats per minute, an invasive arterial BP of 63/30mmHg, and a mean arterial of BP 40mmHg. Her central venous pressure was 6cmH_2_O. Pulse oximetry showed 80% saturation at a fraction of inspired oxygen (FiO2) of 1.0. Generalized crackles were noted bilaterally on auscultation of her lungs. She had cool, mottled skin and a prolonged capillary refill time was noted. Her Glasgow coma score was 6. An abdominal examination revealed no hepatomegaly. Her Pediatric Risk of Mortality III (PRISM-III) score was 21.

The first chest radiograph taken on PICU admission revealed bilateral pulmonary edema with normal heart size (cardiothoracic ratio, 50%). Results of arterial blood gas analysis were as follows: pH, 7.328; partial pressure of oxygen (PO_2_), 99.7mmHg; partial pressure of carbon dioxide (PCO_2_), 37.1mmHg; bicarbonate (HCO_3_^-^), 19.4mmol/L; and oxygen saturation, 99% with FiO_2_ of 1.

Electrocardiography revealed a sinus tachycardia. Two-dimensional and color Doppler echocardiography demonstrated a mild dilated left ventricular with diffuse hypokinesis. The ejection fraction (EF) and shortening fraction (FS) of her left ventricle were 37% and 19%, respectively.

Laboratory investigations revealed the following values: WBC count 13,500 cells/μL, neutrophils 10,180 cells/μL; lymphocytes 2,130 cells/μL; hemoglobin 12.6g/dL; platelet count 188 × 10^3^ cells/μL; prothrombin time 12.7s; activated partial thromboplastin time 35.2s; fibrinogen 4.9g/L; D-dimer 861ng/mL; blood urea nitrogen 3.7mmol/L; creatinine 44μmol/L; blood glucose level 24mmol/L; sodium 125.4mmol/L; potassium 3.7mmol/L; chloride 95mmol/L; calcium 2.02mmol/L; total bilirubin 0.5μmol/L; aspartate aminotransferase 43.31IU/L; alanine aminotransferase 4.05IU/L; lactate dehydrogenase 435IU/L; creatine kinase 382.1IU/L; creatine kinase-MB 44IU/L; troponin T 0.297ng/mL; ammonium 58.7mg/L; lactate 2.94mmol/L; C-reactive protein 0.39mg/L; total protein 68.3g/L; and albumin 36.3g/L.

We confirmed that the child had pulmonary edema and shock as complications of HFMD. The initial management modalities were positive pressure mechanical ventilation support, 10 mL/kg of normal saline given over 30 minutes, and the administration of a dobutamine infusion. Our patient’s arterial BP initially responded well to the dobutamine and was stable for one hour, then her cardiovascular status deteriorated quickly and her BP dropped to 45/30mmHg. An epinephrine infusion was added with titrated doses to stabilize our patient’s BP. Three hours after PICU admission, although her mean arterial BP was stable at 65 to 75mmHg, our patient’s heart rate was consistently high at 210 to 220 beats per minute, and the doses of dobutamine, epinephrine and milrinone were 15μg/kg/min, 0.4μg/kg/min and 0.5μg/kg/min, respectively.

After obtaining parental consent, CVVH was initiated three hours after PICU admission. A double-lumen 8F catheter (Gambro, Hechingen, Germany) was inserted into the femoral vein using the Seldinger technique to establish vascular access. We used the continuous hemofiltration Prisma machine (Gambro), and an M60 hemofilter with polyacrylonitrile (AN69) membranes. To prevent clotting, unfractionated heparin was infused into the blood circuit, with a titrated dose to achieve an activated clotting time of 140 to 160 seconds, or an activated partial thromboplastin time of twice the control. We set the initial blood flow rate at 3mL/kg/min, then increased it to an average of 5mL/kg/min. The substitution fluid (Hemosol BO; Gambro) was infused at a rate of 60mL/kg/h in a pre-diluted manner. Fluid removal from the machine was set at zero initially, and the rate then adjusted based on our patient’s fluids status and central venous pressure. CVVH was sustained for 48 hours without any complication.

**Figure 1:**
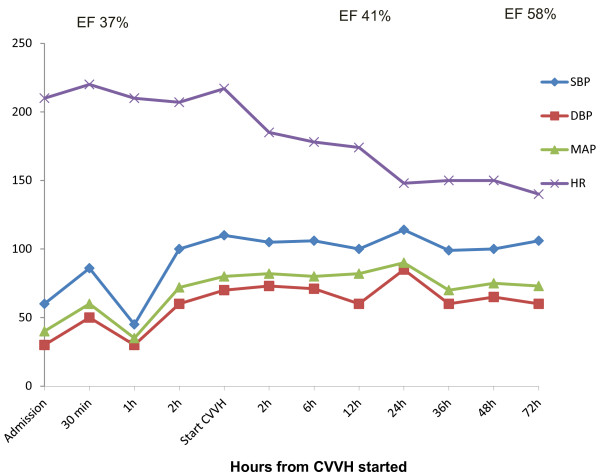
**Hemodynamic parameters prior to and after continuous veno-venous hemofiltration for the first 72 hours.** CVVH: continuous veno-venous hemofiltration; DBP: diastolic blood pressure; MAP: mean blood pressure; SBP: systolic blood pressure; EF: ejection fraction; HR: heart rate; PICU: pediatric intensive care unit.

After starting CVVH, our patient’s hemodynamic status became more stable (Figure 1). Her heart rate gradually decreased, accompanied by a stable arterial BP. Two hours after CVVH commenced, her heart rate was 175 beats per minute, and the dose of epinephrine was tapered to 0.2μg/kg/min. Six hours after starting CVVH, epinephrine was discontinued. Dobutamine was tapered and discontinued after 24 hours of CVVH. Milrinone was continued for 24 hours after CVVH was ceased, with a tapered dose to 0.25μg/kg/min. We discontinued CVVH after 48 hours when our patient’s hemodynamic status was stable with a heart rate of 150 beats per minute and BP of 100/60mmHg. A cardiac ultrasound performed at 72 hours of CVVH revealed an improved left ventricular kinesis, with a left ventricular EF of 58% and FS of 29%. Her PRISM -III score at 24 hours after CVVH was 7. Our patient was successfully extubated 72 hours after CVVH was initiated. A clinical examination revealed no neurological deficit even though brain imaging investigations were not performed. She was discharged home after 11 days of hospitalization without any obvious sequelae. At a three-month follow-up, a full neurological and development assessment revealed no abnormality.

Echocardiography was performed and revealed a normal cardiac function with a left ventricular EF and FS of 65.9% and 34%, respectively.

We measured some cytokines and chemokines in her plasma and effluent fluid using enzyme-linked immunosorbent assay techniques. The plasma level of interferon gamma (IFN-γ), interleukin (IL)-8 and tumor necrosis factor receptor 2 (TNF-R2) measured at the initiation of CVVH were 112.4pg/mL, 141.0pg/mL and 3599.1pg/mL, respectively; and at 24 hours after CVVH were 82.8pg/mL, 85.1pg/mL, and 2376.9pg/mL, respectively. IFN-γ (40.8pg/mL) and IL-8 (7.4pg/mL) were also detected in her ultrafiltration fluid at 24 h after CVVH therapy.

Virological studies performed at the molecular laboratory failed to detect influenza virus, adenovirus, respiratory syncytial virus, Epstein-Barr virus and rhinovirus from various specimens. However, EV71 ribonucleic acid (RNA) was detected from a throat swab and tracheal aspirates by reverse transcription polymerase chain reaction techniques using EV71 specific primers [9]. Blood cultures on hospital and PICU admission were negative.

## Discussion

The outcome of children with EV71-induced fulminant CPF is very poor. The majority of patients with pulmonary edema die within 24 hours of hospitalization. In a group of patients infected with EV71 with complications, Chang *et al*. reported that all 11 patients with pulmonary edema died and nine of them rapidly deteriorated and died within 12 hours of PICU admission, while only one of 38 patients without pulmonary edema died [2]. Recently, with the application of ECLS, the outcome of children with EV71 infection-related cardiopulmonary failure has been modestly improved. Jan *et al*. reported favorable outcomes in six out of eight patients with stage 3a CPF (with hypertension) treated with extracorporeal membrane oxygenation and/or extracorporeal left ventricular support. However, four out of five patients with stage 3b CPF (with hypotension) died and one survived with a poor neurological outcome [5].

The mechanism of pulmonary edema and shock associated with EV71 infection remains unclear. However, studies demonstrated that pulmonary edema may result from the increased pulmonary vascular permeability [10]. Systemic inflammatory response syndrome induced by a mass release of cytokines, a ‘cytokine storm’, may be responsible for increased vascular permeability. Wang *et al*. found that concentrations of plasma IL-10, IL-13 and IFN-γ were all significantly higher in patients with pulmonary edema than those without [3]. In addition, they also found that several chemokines in plasma, including IL-8, IFN-γ-induced protein-10, monocyte chemoattractant protein-1 and monokine induced by IFN-γ, were significantly higher in patients with pulmonary edema than in those with uncomplicated brainstem encephalitis [4].

The other possible pathophysiological mechanism of EV71-related fulminant CPF is massive release of catecholamines, a ‘catecholamine storm’, as a result of brainstem encephalitis [10]. The impact of CRRT on the catecholamine clearance in patients with severe EV71 infection is unknown and requires further investigation. However, Bellomo *et al*. found that, in patients with hemodynamic instabilities requiring inotropic or vasopressor support, continuous hemodiafiltration could remove catecholamines but did not significantly affect plasma catecholamine levels and had no impact on hemodynamic status [11]. Similar findings were found in critically ill patients with acute renal failure: CRRT did not produce significant catecholamine losses [12]. In our patient, the epinephrine dose could be tapered after starting CRRT therapy. Therefore, it was unlikely that the hemodynamic improvement in our patient was the benefit of catecholamine clearance.

The application of CRRT has improved the outcomes of critically ill patients, especially those with multiple organ failure due to severe sepsis and septic shock [6]. CRRT has been proposed to improve the outcomes of patients with systemic inflammatory response syndrome and sepsis. CVVH increases the removal of inflammatory mediators or a pathogen’s toxin from the blood by a convective mechanism and/or by adsorption to the membrane of the hemofilter. The effectiveness of the therapy depends on the time of initiation and the dose of ultrafiltration [13]. Early CVVH improved the hemodynamic status of adult patients with septic shock [7]. Peng *et al*. reported a marked reduction of plasma levels of IFN-γ, and IL-1, -2, -4, -10 and −13 in septic patients after CVVH treatment. In addition, the clinical status of patients treated with CVVH improved significantly compared with those without CVVH treatment [8].

To the best of our knowledge, this is the first case reported in the medical literature about the application of CVVH in management of EV71-related cardiopulmonary failure. CVVH was chosen as the initial mode of CRRT for our patient because the middle molecular solutes such as cytokines are best cleared from the plasma by the convective mechanism of CVVH [13]. In addition, CVVH has been documented as an effective mode of CRRT for sepsis and septic shock patients in previous studies [7,8]. We started CVVH early when our patient’s hemodynamic status was not stable, and used a high-volume hemofiltration rate (60mL/kg/h). Our patient’s hemodynamic status improved quickly after the initiation of CVVH. The inotropes were tapered and discontinued, and her cardiac function improved markedly after CVVH therapy. The change in her plasma cytokines level and detection of cytokines in the effluent fluid after 24 hours of CVVH therapy indicated that cytokines were removed from the blood.

## Conclusions

We conclude that CVVH may be an effective therapy in the management of fulminant CPF due to EV71 infection. The beneficial effect of CVVH may depend on the timing of the initiation of therapy and the dose of hemofiltration. Further studies are needed to confirm the benefit of this therapy.

## Consent

Written informed consent was obtained from the patient’s parents for publication of this case report and accompanying images. A copy of the written consent is available for review by the Editor-in-Chief of this journal.

## Abbreviations

BP, Blood pressure; CPF, Cardiopulmonary failure; CRRT, Continuous renal replacement therapy; CVVH, Continuous veno-venous hemofiltration; ECLS, Extracorporeal life support; EF, Ejection fraction; FS, Shortening fraction; EV71, Enterovirus 71; FiO2, Fraction of inspired oxygen; HFMD, Hand, foot and mouth disease; IFN-γ, Interferon gamma; IL, Interleukin; TNF-R2, Tumor necrosis factor receptor 2; PICU, Pediatric intensive care unit; PRISM-III, Pediatric Risk of Mortality III; WBC, White blood cell.

## Competing interests

The authors declare that they have no competing interests.

## Authors’ contributions

PP was the major contributor to the case treatment and presentation, conducted the literature review and wrote the manuscript. HD and SC were involved in the patient care and collected the data regarding the patient’s history and clinical course as well as the trends in vital parameters. TBP performed virological tests and measured cytokines level. TVP and TN reviewed the manuscript and were involved in the patient care. LN provided the concept for the study and was a major contributor in the manuscript revision. All authors read and approved the final manuscript.
